# A Case of X-Linked Hypophosphatemic Rickets with Dentin Dysplasia in Mandibular Third Molars

**DOI:** 10.3390/children9091304

**Published:** 2022-08-28

**Authors:** Rena Okawa, Masakazu Hamada, Misato Takagi, Saaya Matayoshi, Kazuhiko Nakano

**Affiliations:** 1 Department of Pediatric Dentistry, Osaka University Graduate School of Dentistry, Osaka 565-0871, Japan; 2 Department of Oral and Maxillofacial Surgery II, Osaka University Graduate School of Dentistry, Osaka 565-0871, Japan

**Keywords:** X-linked hypophosphatemic rickets, dentin dysplasia, mandibular third molar, oral management

## Abstract

X-linked hypophosphatemic rickets (XLH) is a disease characterized by impaired bone mineralization, and its dental features include gingival abscesses and large pulp spaces due to dentin dysplasia. A 20-year-old woman with XLH was referred to oral surgery for extraction of mandibular third molars. She was diagnosed with XLH at approximately 1 year of age and was treated thereafter. There was no history of gingival abscesses, and panoramic radiographic and computed tomographic examinations revealed no evidence of dentin dysplasia. However, histopathological examination of the extracted teeth showed dentin dysplasia, including interglobular dentin. In this XLH patient, dentin dysplasia was revealed histologically even though no obvious abnormality was found on visual and radiographic examinations. These findings suggest that in patients with XLH, oral management must take dentin dysplasia of the permanent teeth into consideration even if the patient’s general condition is well controlled with conventional therapy.

## 1. Introduction

X-linked hypophosphatemic rickets (XLH) is a rare skeletal disease and the most common form of inherited rickets [1,2,3]. The prevalence of XLH is estimated to be 1/20,000 people in North America and Japan, and no substantial race-related difference in the incidence of XLH has been identified [2]. XLH is caused by mutation of the *PHEX* gene (phosphate-regulating gene with homology to endopeptidases on the X chromosome) [4,5]. XLH is known to cause the following systemic symptoms due to inhibition of phosphorus reabsorption by *PHEX*: severe deformities of the lower extremities such as O- and X-legs, growth disturbances such as short stature, bone and muscle pain, and decreased quality of life [4,5]. Because XLH is a multisystem disease, it requires a multidisciplinary approach in specialized fields [4,5].

*PHEX* is also expressed in odontoblasts, resulting in dental characteristics including dentin dysplasia in patients with XLH [6,7]. Histopathologically, dentin is characterized by numerous intercalated dentin globules, enlarged dentin protuberances, and irregular dentin tubules, which have been reported frequently in primary teeth but less frequently in permanent teeth [8,9,10,11]. When dysplastic dentin is exposed due to occlusion and wear of the enamel, the pulp is infected by oral bacteria, and a spontaneous periapical abscess may form at the root apex of an apparently healthy tooth without dental trauma or caries [8,10,12,13,14].

Conventional therapy for systemic treatment of XLH is phosphorus correction and calcium administration [4]. Some reports have examined the effect of conventional therapy on the dental symptoms of Hyp mice (a mouse model of XLH) [15,16,17]. However, the dental effects of conventional therapy on humans are unknown [14,18].

In the present case, we had the opportunity to extract the third molars of a patient with XLH and examine them histologically and radiologically. We discuss the dental effects of conventional therapy for XLH, focusing on the timing of tooth formation and systemic treatment.

## 2. Case Presentation

**Figure 1:**
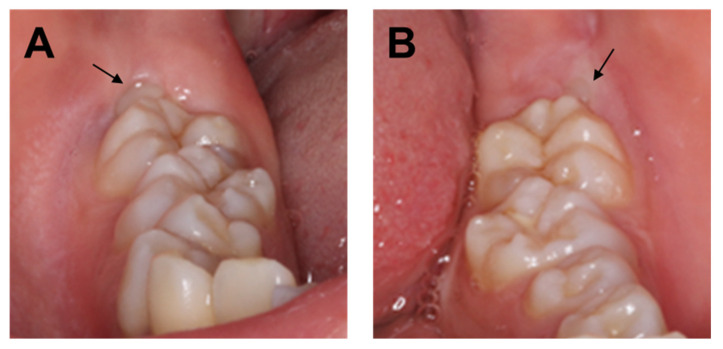
(**A**) Intraoral photograph of the right mandible taken at the initial visit. (**B**) Intraoral photograph of the left mandible taken at the initial visit. Arrows indicate mandibular third molars.

**Figure 2:**
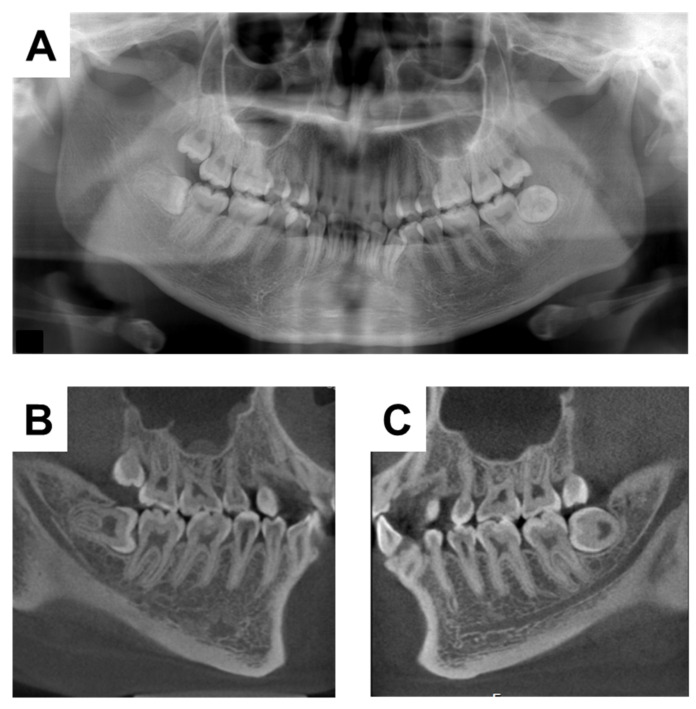
X-ray examinations at the initial visit. (**A**) Orthopantomograph. (**B**) Computerized tomographic image of the right side. (**C**) Computerized tomographic image of the left side.

A 20-year-old woman with XLH was referred to the Department of Oral and Maxillofacial Surgery at Osaka University Dental Hospital for evaluation of her mandibular third molars. She had been diagnosed with XLH at the age of approximately 1 year, after being referred to a pediatrician with a chief complaint of O-legs. She was diagnosed with XLH based on the presence of rachitic changes in X-ray images, a high alkaline phosphatase concentration, hypophosphatemia, hypocalcemia, and clinical signs such as bone deformities [19]. Treatment with 1 α-OH-D_3_ (Alfarol^®^, Chugai Pharmaceutical Co. Ltd., Tokyo, Japan) was started, and at 6Y6M, sodium phosphate (Phosribbon^®^, Zeria Pharmaceutical Co. Ltd., Tokyo, Japan) was added; these two drugs controlled her condition. Her calcium was controlled at a higher level and inorganic phosphorus at a lower level (Table 1). There was no history of gingival abscesses in either the primary or permanent teeth. The patient had received periodic oral management to prevent dental caries, including oral hygiene control and fluoride application. The bilateral mandibular third molars were impacted. No gingival swelling was observed around the third molars, and the crowns of the other erupting teeth appeared normal at the time of the oral examination (Figure 1). There were no obvious dental caries, and there was no history of tooth restoration, nor root canal treatment on orthopantomography (Figure 2A). Additionally, the pulp chamber was not particularly wide, with no obvious signs of dentin dysplasia (Figure 2A). Computed tomographic examinations showed that the mandibular third molars were positioned horizontally and in close proximity to the mandibular canal (Figure 2B,C). On both sides, the mandibular third molars had partially exposed crowns and were judged to require extraction.

**Figure 3:**
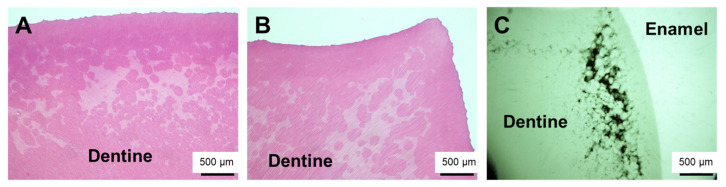
Histological examination of the extracted tooth showed characteristic wide globular dentin. (**A**) Right mandibular third molar (stained with H and E). (**B**) Left mandibular third molar (stained with H and E). (**C**) Right mandibular third molar (contact micrograph image of ground section).

During the surgical extraction, cutting of the bone and teeth did not seem abnormal. Because there was a history of XLH, we decided to perform histopathological examination of the extracted teeth for future oral management. The crown of the tooth was fixed in 10% neutral buffered formalin and then hemi-sectioned, with one half subjected to histological analysis. First, demineralization with 0.5 M EDTA at 4 °C and conventional processing for paraffin embedding were performed. The specimen was then cut into 5 µm thick sagittal sections, mounted on silane-coated slides, and subjected to hematoxylin and eosin (H&E) staining. Histopathological examination of the decalcified section showed tubular defects and interglobular dentin (Figure 3A,B). The other half of the right molar specimen was conventionally embedded with methyl methacrylate. The embedded specimen was cut with an ultra-low-speed saw. It was subsequently reduced to a final thickness of approximately 150 µm by grinding. The ground section was exposed to soft X-rays generated by a contact microradiographic device (Sofron Type SRO-405; Sofron Co. Ltd., Tokyo, Japan) operated at 11 kV and 5 mA for 10 min. The absorption was registered on Professional Special Holographic Film SO-181 (Eastman Kodak Company, Rochester, NY, USA). Contact microradiograph imaging of a ground section also revealed tubular defects from the enamel–dentin junction to the pulp in the dentin and interglobular dentin (Figure 3C).

**Figure 4:**
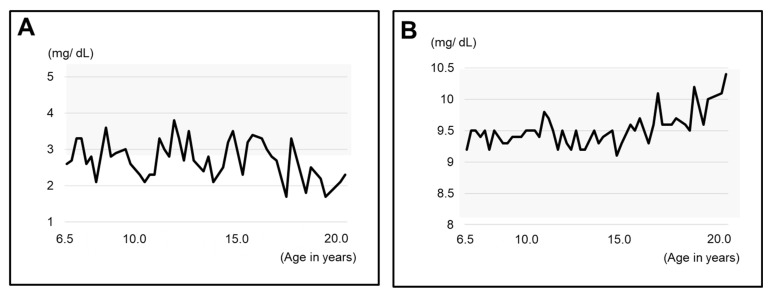
Blood test results related to XLH since the age of 6Y6M. (**A**) Inorganic phosphorus (IP). (**B**) calcium (Ca). The gray zone indicates the range of standard values.

We were able to obtain blood test results from the age of 6Y6M, which showed that calcium was well controlled at a higher level and inorganic phosphorus at a lower level (Figure 4A,B).

**Figure 5:**
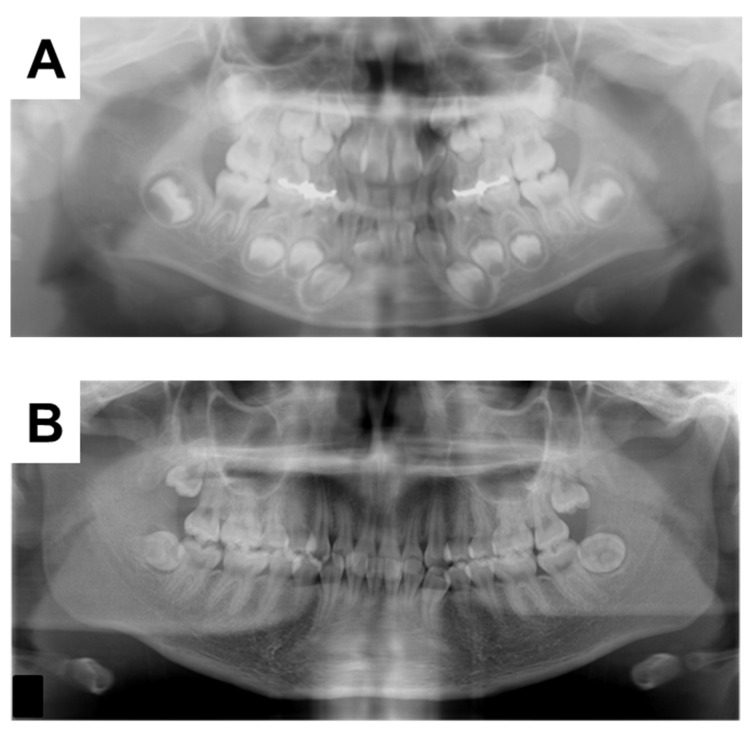
Orthopantomographs taken during the follow-up period at (**A**) 6Y6M and (**B**) 17Y5M.

**Figure 6:**
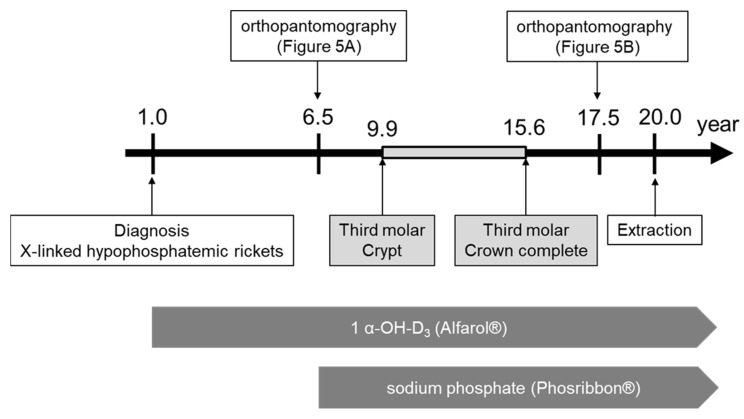
Schema of treatment progress.

The 6Y10M orthopantomograph showed that the first molars had erupted and the tooth embryos of the second molars were visible (Figure 5A). We estimated the dental age on the basis of the development stage of the permanent teeth using the method of Kuremoto, which was established in Japanese subjects [20]. Kuremoto reported age medians in years for 12 tooth formation stages for boys and girls separately, as well as for the maxilla and mandible. In this study, a single pediatric dentist assessed the formation stage of all the permanent teeth using orthopantomography. The formation stages were then converted to chronological age, and the average of those chronological ages was considered to be the dental age of the patient. The 6Y6M orthopantomography assessment was similar to the chronological age at 6Y4M (Table 2), indicating that the rate of calcification of the permanent teeth was similar to that of healthy children before the start of treatment with Phosribbon^®^. In addition, the tooth germs of the mandibular third molars could not be discerned. In the 18Y3M orthopantomograph, the third molars were under root formation (Figure 5B). Despite phosphorus levels being corrected throughout the duration of the formation of the mandibular third molar crowns, dentin dysplasia was observed in the present case (Figure 6).

## 3. Discussion

XLH is a rare genetic disorder caused by mutation of *PHEX* [4,5,21]. Because *PHEX* is located on the X chromosome, random inactivation of the X chromosome in females is predicted to result in a milder phenotype than in males with complete loss of *PHEX* [10,22,23]. In the present case, there were no typical clinical signs of dentin dysplasia such as periapical abscesses, and the dental symptoms were considered mild.

It was reported that the tooth crypt of the lower third molar emerges at 9.9 ± 1.4 years in Japanese females [20]. In a previous report on the relationship between XLH treatment and tooth formation, one patient started conventional therapy at the age of 2Y11M, but the treatment was temporarily interrupted during puberty, which coincided with the period of tooth root dysplasia [8]. In the present case, the patient was diagnosed with XLH at the age of 1 year. Treatment with 1 α-OH-D_3_ (Alfarol^®^) was started, and at 6Y6M, sodium phosphate (Phosribbon^®^) was added, after which the condition was controlled. There were no episodes of discontinuation of medication. The results of blood tests since the age of 6Y6M showed that calcium was controlled at a high level and inorganic phosphorus at a low level, indicating good control. These results show that even though XLH was well controlled, dentin dysplasia was still observed. It is not clear whether the good control lessened the severity, but it did not completely suppress dentin dysplasia. The dentin dysplasia in Hyp mice is not influenced by the short-time normalization of the serum phosphate concentration [15]. Teeth are embryologically similar to bone; however, teeth do not undergo remodeling. Therefore, we believe that the effects of conventional treatments may be different for bone and teeth.

It has been reported that there is no way to determine how extensively dentin and enamel formation is affected by XLH [10]. In the present case, dentin dysplasia was revealed histologically even though no obvious abnormality was found on visual and radiographic examinations. This suggests that in patients with XLH, oral management must take dentin dysplasia of the permanent teeth into consideration, even if the patient’s general condition is well controlled with conventional therapy. Pulp infection by oral bacteria via exposed hypomineralized dentin is caused by fracture, abrasion, or caries of enamel. Preventive treatments such as fissure sealants or enamel filling are recommended.

## 4. Conclusions

The presence of dentin dysplasia when there is no apparent evidence on X-ray or intraoral examination, as in this case, highlights the high risk of pulp infection via hypomineralized dentin and the importance of early treatment and preventive care in XLH patients.

## Figures and Tables

**Table 1:** Results of blood analysis at 20 years old.

Item	Abbreviation	Value	Standard Value
Inorganic phosphorus	IP	2.1 mg/dL	2.9–4.8 mg/dL
Calcium	Ca	10.1 mg/dL	8.6–10.3 mg/dL
Alkaline phosphatase	ALP	113 U/L	38–113 U/L
Aspartate aminotransferase	AST	26 U/L	≦40
Alanine aminotransferase	ALT	35 U/L	≦40
Albmin	Alb	4.4 g/dL	3.6–4.7 g/dL
Urea nitrogen	UN	14 mg/dL	7–22 mg/dL
Creatinin	Cre	0.57 mg/dL	0.5–0.9 mg/dL
e glomerular filtration rate creat	eGFR creat	112.2	
25-hydroxyvitamin D3	25-(OH)D3	14 ng/mL	20–60 ng/mL
1,25-dihydroxyvitamin D3	1,25-(OH)2D3	61 pg/mL	61 pg/mL
Parathyroid hormone	PTH	85.8 pg/mL	<20
Fibrosis 4 index	FiB4 index	0.38	

**Table 2:** The dental age of each tooth at 6Y6M.

**Upper**	7.0	6.8	6.9	6.8	7.2	6.7	6.8	6.8	5.6	6.8	6.8	6.9	6.8	7.0
**Tooth**	7	6	5	4	3	2	1	1	2	3	4	5	6	7
**Lower**	6.2	6.3	6.4	5.4	5.6	5.7	6.5	6.5	6.4	5.6	5.4	6.4	6.3	6.2

## Data Availability

Not applicable.
